# Supplementary material for: Brain-immune interactions generate pathogen-specific sickness states
Source: bioRxiv. 2025 Dec 8:2025.12.06.692770. Preprint. [Version 1] doi: 10.64898/2025.12.06.692770 (PMC12713759; doi:10.64898/2025.12.06.692770)

bioRxiv preprint doi: https://doi.org/10.64898/2025.12.06.692770; this version posted December 8, 2025. The copyright holder for this preprint (which was not certified by peer review) is the author/funder, who has granted bioRxiv a license to display the preprint in perpetuity. It is made available under aCC-BY 4.0 International license.

Figures:

Figure S1. Related to Figure 1.

(A) Normalized confusion matrices showing accuracy of random forest classifier for predicting sickness state based on 42 measured serum cytokines. F1 = 0.525 +/- 0.085. (B) Normalized confusion matrices showing accuracy of random forest classifier for predicting sickness state based on serum cytokines shown in Fig. 1B F1= 0.597 +/- 0.158. (C) z-scored concentration of 42 serum cytokines measured by multiplexed ELISA in males and females during different sickness states. (D) Temperature in male FVB mice during different sickness states (E) Body temperature in female FVB mice during different sickness states (F) Food intake in male FVB mice during different sickness states over 4h (G) Food intake in FVB female mice during different sickness states over 4h (H) Distance traveled by male FVB mice during different sickness states over 10 minutes (I)

bioRxiv preprint doi: https://doi.org/10.64898/2025.12.06.692770; this version posted December 8, 2025. The copyright holder for this preprint (which was not certified by peer review) is the author/funder, who has granted bioRxiv a license to display the preprint in perpetuity. It is made available under aCC-BY 4.0 International license.

Average locomotor velocity of female FVB mice during different sickness states over 10 minutes (J) VO_2 in B6 male mice during different sickness states (K) VCO_2 in B6 male mice during different acute sickness states (L) VO_2 in B6 female mice during different acute sickness states (M) VCO_2 in B6 female mice during different sickness states (N) RER in B6 male mice during DSS challenge (O) RER in B6 female mice during DSS challenge. $p<0.05$* $p<0.01$** $p<0.001$*** $p<0.0001$**** based on ordinary one-way ANOVA with Dunnett's multiple comparisons test. All measurements taken at 4h post-injection (acute models) or day 3 (DSS).

Figure S2. Related to Fig. 2

(A) (*Ad libitum* social interaction in C57BL/6 female mice recorded over 10 minutes.B) summary of individual social behaviors quantified in (A). (C) social rebound in B6 female mice recorded over 10 minutes. (D) summary of individual behaviors quantified in (C). (E-

bioRxiv preprint doi: https://doi.org/10.64898/2025.12.06.692770; this version posted December 8, 2025. The copyright holder for this preprint (which was not certified by peer review) is the author/funder, who has granted bioRxiv a license to display the preprint in perpetuity. It is made available under aCC-BY 4.0 International license.

J) subset of USV acoustic features computed using WarbleR Heat maps represent z-scored data. All measurements taken at 4h post-injection (acute models) or day 3 (DSS).

Figure S3. Related to Fig. 3

(A) Graphical heat map of average FOS intensity across coronal sections in all sickness states, normalized to saline controls. (B-F) Hierarchical clustering of FOS intensity normalized to saline controls in brain areas grouped by functional categories, separated by sex. Acronyms of brain areas shown in (A) listed in Table S1.

bioRxiv preprint doi: https://doi.org/10.64898/2025.12.06.692770; this version posted December 8, 2025. The copyright holder for this preprint (which was not certified by peer review) is the author/funder, who has granted bioRxiv a license to display the preprint in perpetuity. It is made available under aCC-BY 4.0 International license.

Figure S4. Related to Fig. 4:

Validation of feature selection and model stability. (A) Quantitative validation of clustering enhancement: Mean silhouette score and Calinski-Harabasz index for full (641 areas) and Boruta-selected (36/25 areas) data. The Boruta feature selection significantly improved data partition. The silhouette distance became more positive (or less negative) in both sexes (left: males, right: females) after selection. The Calinski-Harabasz index showed a dramatic increase from 3.18 to 17.37 in males and 2.43 to 9.45 in females. (B) Elastic net hyperparameter selection: plots illustrating the Mean squared error (MSE) dependence on the Elastic net regularization hyperparameters, λ and α, used during model training. The choice of λ and α was optimized for robustness in handling high-dimensional, potentially correlated FOS data. Prediction probabilities and feature importance in males (C-E) and females (F-H): Prediction probabilities for individual test samples across the six sickness states in males (C) and females (F), showing high prediction probabilities were consistently assigned to the true sickness state. Plots showing the distribution of coefficients across all cross-validation iterations and sickness states, providing insight into the stability and consistency of each region's predictive influence in males (D) and females (G). Heatmaps depicting a hybrid metric, defined as $|\text{SHAP}|*\beta$, combining the magnitude of SHAP-style contributions with the stability of the model coefficients for males (E) and females (H).

bioRxiv preprint doi: https://doi.org/10.64898/2025.12.06.692770; this version posted December 8, 2025. The copyright holder for this preprint (which was not certified by peer review) is the author/funder, who has granted bioRxiv a license to display the preprint in perpetuity. It is made available under aCC-BY 4.0 International license.

Figure S5. Related to Fig. 5

(A) Number of differentially-expressed genes identified by bulk RNAseq in the POA and PVN across conditions. (B) Top DEGs in each condition assessed by bulk RNAseq (C&D) expression genes of interest assessed by bulk RNAseq. (E) Correlation between MERFISH and scRNA-seq counts from the Allen Brain Institute (hypothalamic region). (F) Correlation between MERFISH counts from two PBS-treated animals. (G) MERFISH sample-to-sample correlation. (H) Example spatial localizations of MERFISH genes (above) and corresponding Allen Brain in situ hybridization (ISH) data in the mPOA and PVN. (I) Label transfer from P65 hypothalamus Multiome snRNA. Heatmap shows mean label probability applied to MERFISH data. Only a subset of labels are shown. (J) Summary of all neuronal cell-types annotated using MERFISH and correspondence to known functions. Orange circles indicate cell-types involved in homeostatic behaviors. Purple circles represent cell-types involved in social behavior. Scale = 500μm.

bioRxiv preprint doi: https://doi.org/10.64898/2025.12.06.692770; this version posted December 8, 2025. The copyright holder for this preprint (which was not certified by peer review) is the author/funder, who has granted bioRxiv a license to display the preprint in perpetuity. It is made available under aCC-BY 4.0 International license.

Figure S6. Related to Fig. 6

(A) Cell type-specific gene expression changes in the POA (B) Cell type-specific gene expression changes in the PVN (C) Spatial location of upregulated immune genes in the POA (D) Spatial location of upregulated immune genes in the PVN. Scale = 500μm

bioRxiv preprint doi: https://doi.org/10.64898/2025.12.06.692770; this version posted December 8, 2025. The copyright holder for this preprint (which was not certified by peer review) is the author/funder, who has granted bioRxiv a license to display the preprint in perpetuity. It is made available under aCC-BY 4.0 International license.

Supplementary Tables

Supplementary Table 1. Activated brain areas during sickness. Related to Fig. 3 and Fig. S3.

This table lists the acronyms and full names of brain areas shown in coronal section heatmaps in Figures 3 and S3.

Supplementary Table 2. Top Boruta-selected brain regions contributing to classification in males. Related to Fig. 4.

This table lists the 36 brain regions selected via Boruta feature selection (100 repetitions, top 5%) from the original 641 regions for male animals. These regions were used as input for downstream multinomial logistic regression with elastic net regularization. The table includes region names and final area identifiers after standardization. These features showed class-specific predictive value in distinguishing among sickness states (PBS, LPS, PIC, PLA, STAG, DSS).

Supplementary Table 3. Top Boruta-selected brain regions contributing to classification in females. Related to Fig. 4.

This table contains the 25 brain regions selected from the full feature space using Boruta feature selection (100 repetitions, top 5%) for female animals. These were the features used in the multinomial elastic net classifier to predict class membership across inflammation conditions.

Supplementary Table 4: Sequences Associated with MERFISH. Related to Fig. 5

The 'MERFISH Probe Sequences' contains a name and sequence for each of the template oligonucleotides used to create the MERFISH probes associated with combinatorial barcodes. The 'Sequential FISH Sequences' sheet contains the name and sequence of oligonucleotide probes used to target the gene and isoform id listed in the name for genes targeted with non-combinatorial, sequential staining. The 'NBCTL1 Codebook' and 'NBIPL1 Codebook' sheets contain the gene names, isoform ids, and

bioRxiv preprint doi: https://doi.org/10.64898/2025.12.06.692770; this version posted December 8, 2025. The copyright holder for this preprint (which was not certified by peer review) is the author/funder, who has granted bioRxiv a license to display the preprint in perpetuity. It is made available under aCC-BY 4.0 International license.

binary barcode associated with each gene targeted by each of these barcode sets. Entries starting with 'Blank' represents barcodes not assigned to an RNA. The 'Codebook-Readout Mapping' sheet contains the bit number associated with each readout sequence for each library. The 'Other Oligo Sequences' sheet contains the name, sequence, and purpose for all other oligonucleotides used in MERFISH, library amplification, and sequential FISH.

Supplementary Table 5: Gene Expression in Clusters Identified in MERFISH. Related to Fig. 5. The average log-normalized expression for all measured genes in each of the identified cell populations for mice treated with saline. Gene names are listed in the first row and cell type names are listed in the first column.

Supplementary Table 6: Properties of Induced Gene Expression in Sickness States Measured with MERFISH. Related to Fig. 6

The 'Num DEG Cell Types' sheet contains the number of cell types, split by Neuronal or Non-neuronal, in which each of the listed genes was differentially expressed. The 'Delta Moran's I' sheet provides the measured differences between the Moran's I averaged across all measured slices in each sickness state listed and that measured for the samples treated with PBS.

bioRxiv preprint doi: https://doi.org/10.64898/2025.12.06.692770; this version posted December 8, 2025. The copyright holder for this preprint (which was not certified by peer review) is the author/funder, who has granted bioRxiv a license to display the preprint in perpetuity. It is made available under aCC-BY 4.0 International license.

Sullivan et al Figure S1

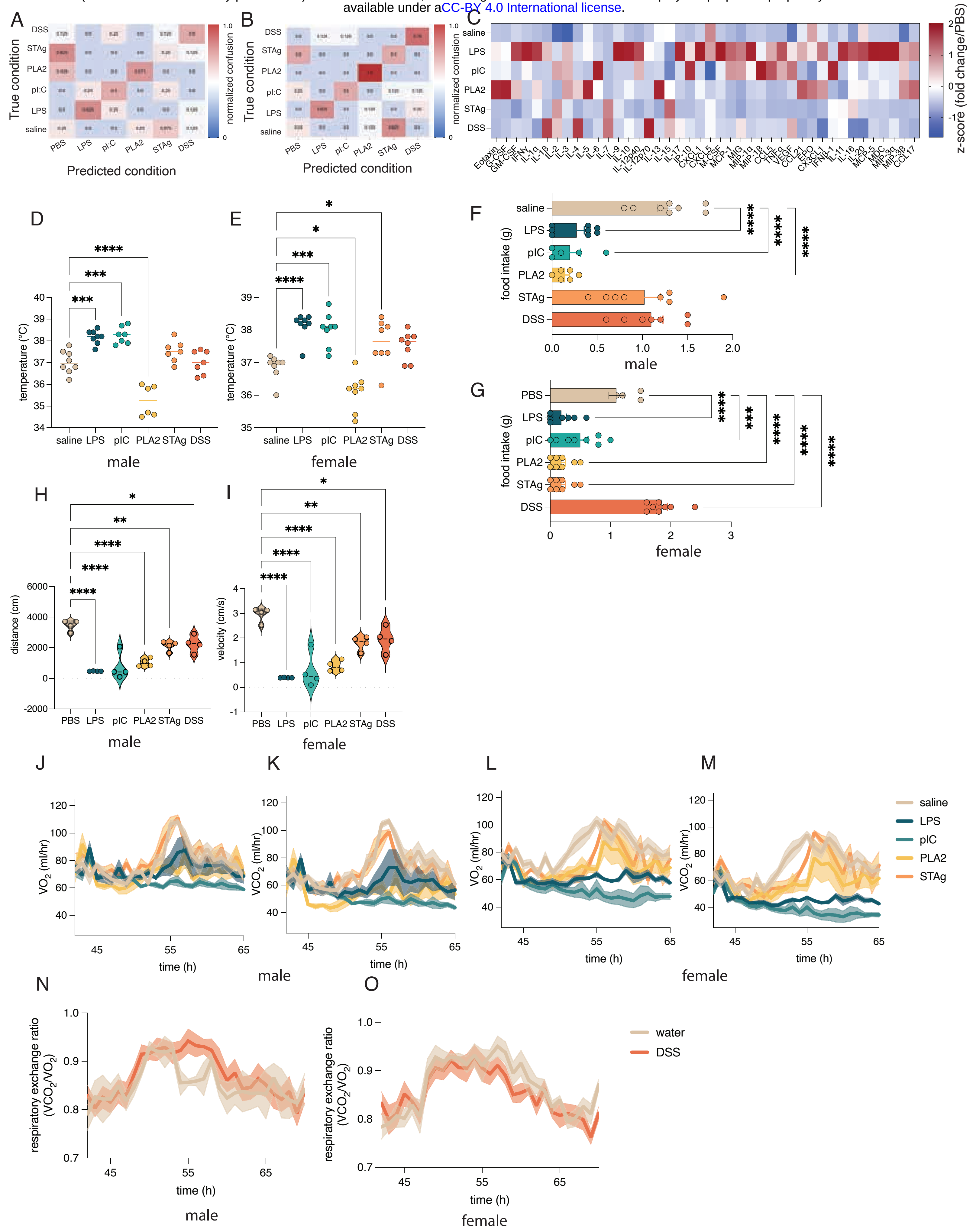

bioRxiv preprint doi: https://doi.org/10.64898/2025.12.06.692770; this version posted December 8, 2025. The copyright holder for this preprint (which was not certified by peer review) is the author/funder, who has granted bioRxiv a license to display the preprint in perpetuity. It is made available under aCC-BY 4.0 International license.

Sullivan et al Figure S2

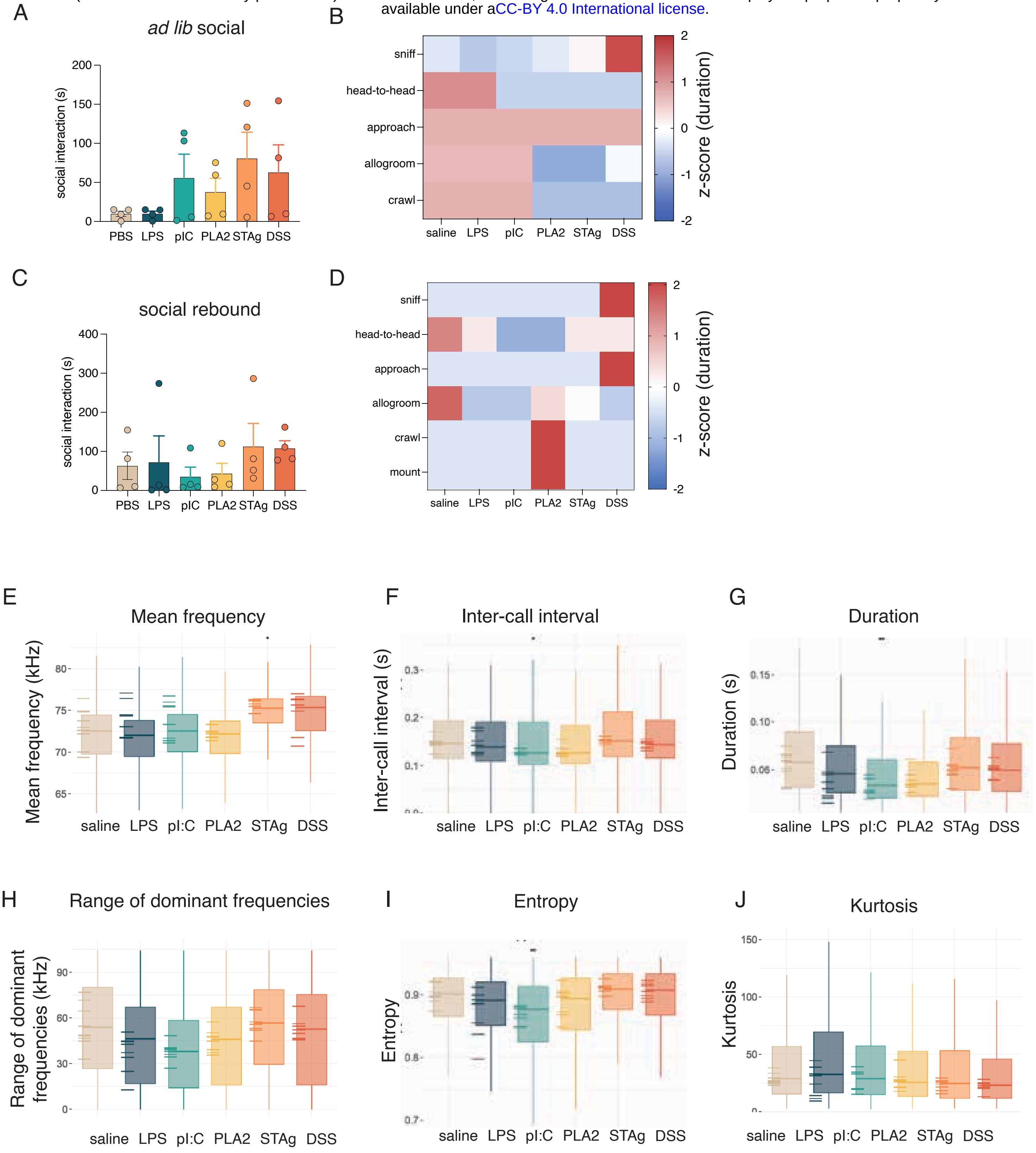

bioRxiv preprint doi: https://doi.org/10.64898/2025.12.06.692770; this version posted December 8, 2025. The copyright holder for this preprint (which was not certified by peer review) is the author/funder, who has granted bioRxiv a license to display the preprint in perpetuity. It is made available under aCC-BY 4.0 International license.

Sullivan et al Figure S3

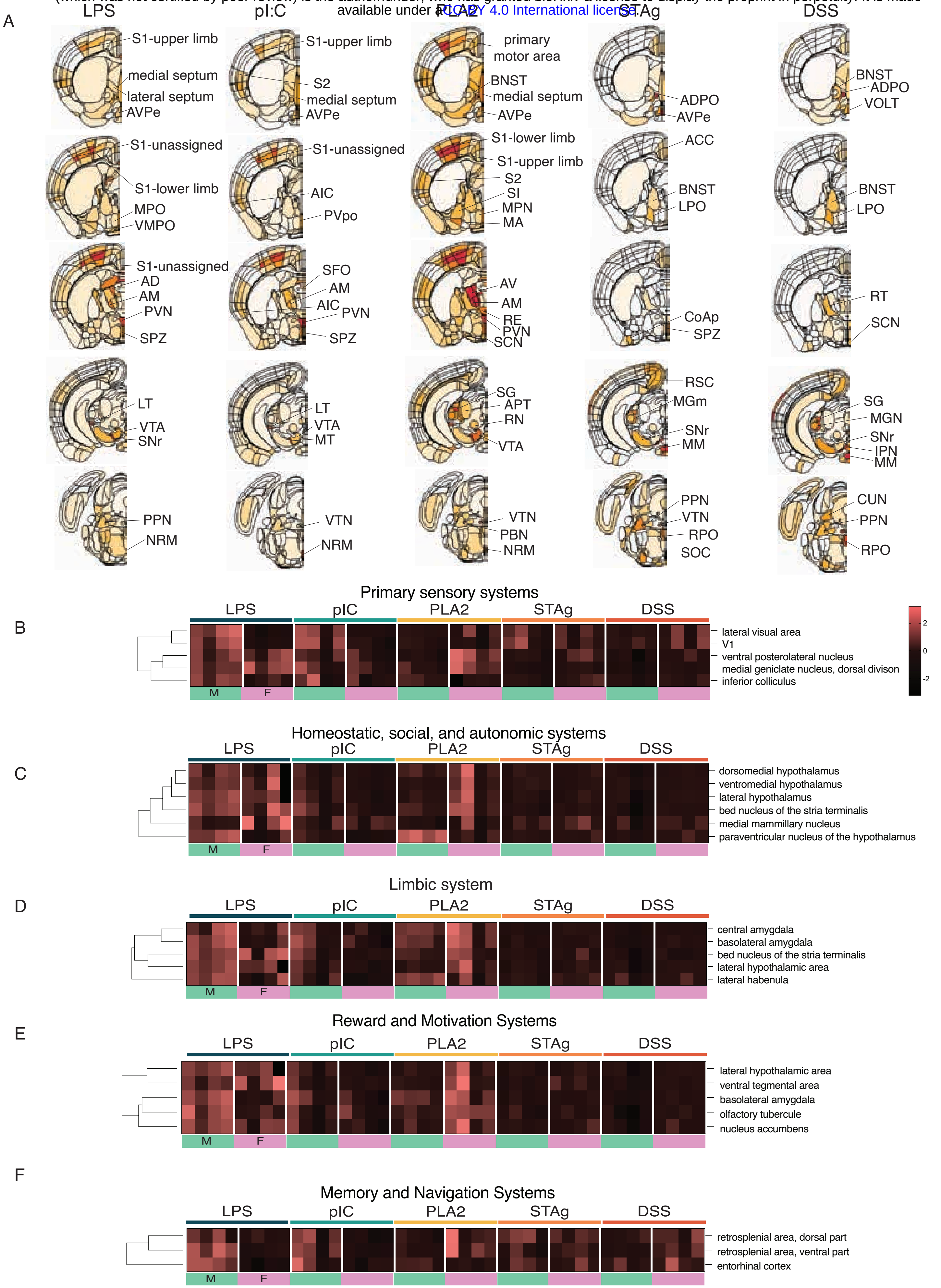

bioRxiv preprint doi: https://doi.org/10.64898/2025.12.06.692770; this version posted December 8, 2025. The copyright holder for this preprint (which was not certified by peer review) is the author/funder, who has granted bioRxiv a license to display the preprint in perpetuity. It is made available under aCC-BY 4.0 International license.

Sullivan et al Figure S4

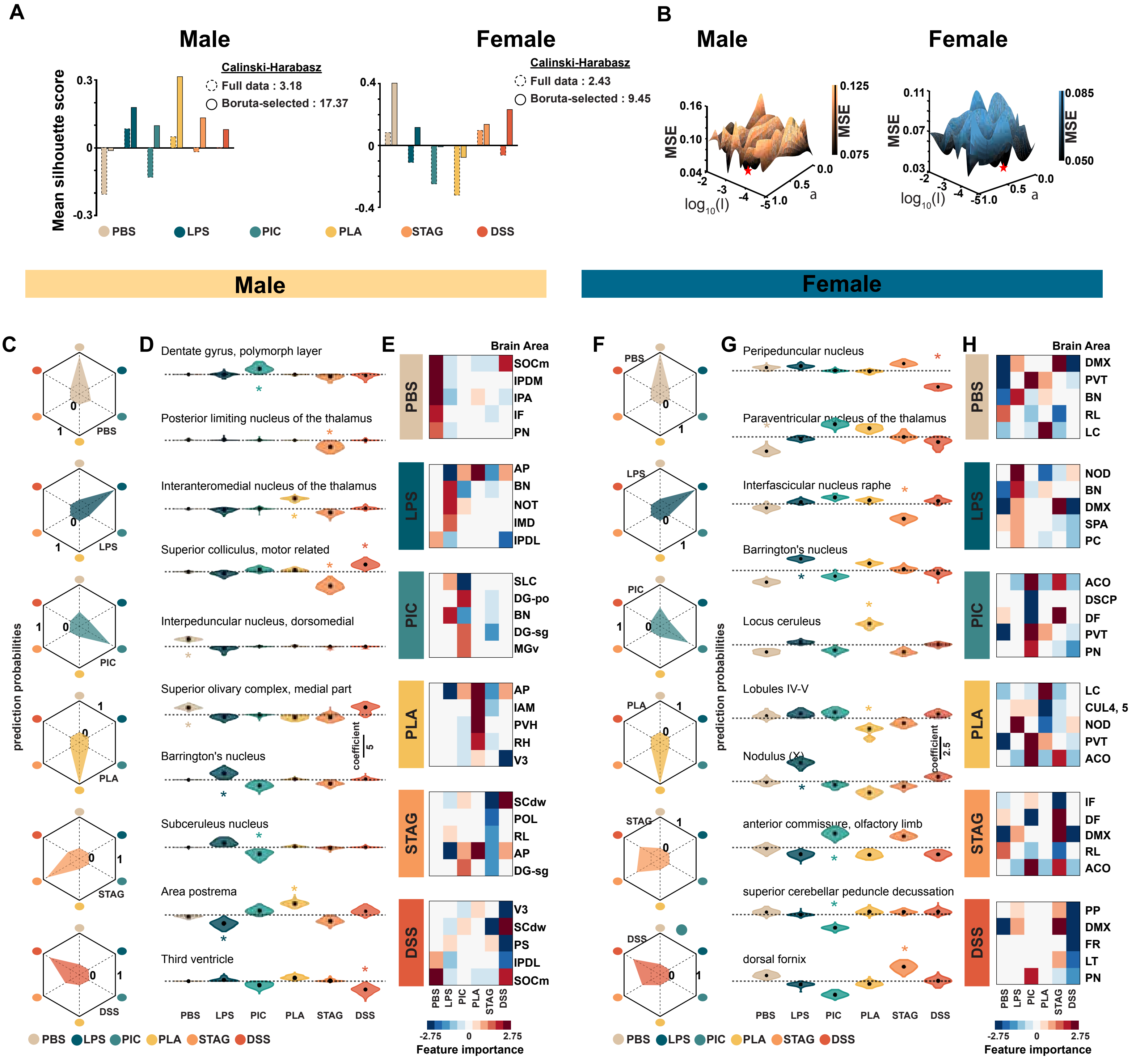

bioRxiv preprint doi: https://doi.org/10.64898/2025.12.06.692770; this version posted December 8, 2025. The copyright holder for this preprint (which was not certified by peer review) is the author/funder, who has granted bioRxiv a license to display the preprint in perpetuity. It is made available under aCC-BY 4.0 International license.

Sullivan et al Figure S5

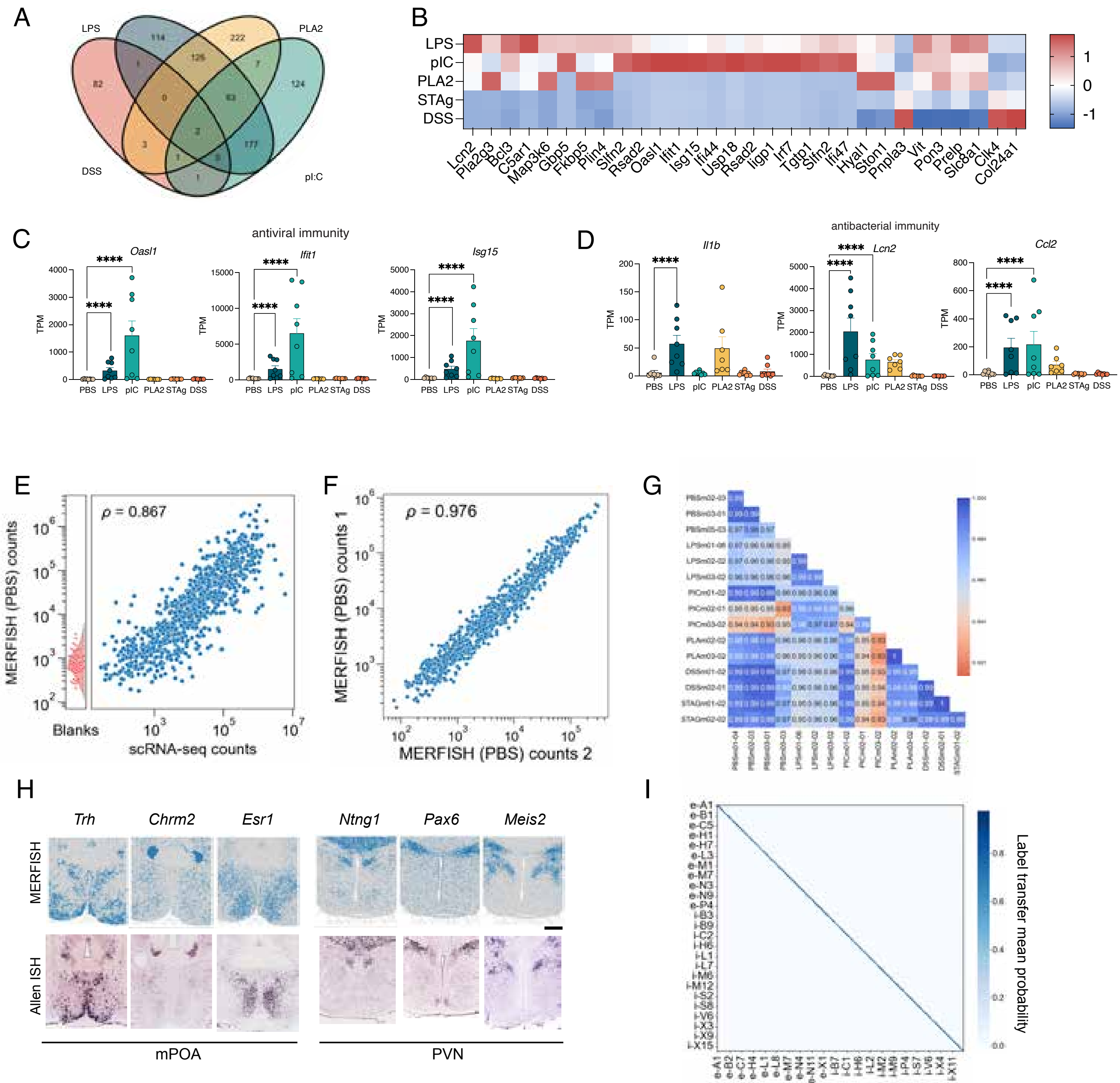

J

Excitatory neuron types	
**AvPe / MnPO**	**MPN anterior**
e-A1:AvPe/MnPO (Brs3/Vglut2)	e-M1:MPN/Pe (Mc4r/C1ql2)
e-A2:AvPe/MnPO (Etv1/Agtr1a)	e-M2:MPN/Pe/VMPO (Nts/C1ql2)
e-A3:AvPe/VMPO (Sncg/Opn5)	e-M3:MPN/VMPO (Calcr/C1ql2)
e-A4 (Nms)	e-M4:MPN/VMPO (Pth2r/Adamts9)
**BAC / BNST**	e-M5:MPN/VMPO (Npy2r/Hmx2)
e-B1:BAC (Ebf2/Cck)	e-M6:MPN/StHy (Trhr/Sox1ot)
e-B2:BNST (Ebf2/Ptprm)	e-M8:MPN (Sox14/Ust)
**AHN / RCH / LHA / ZI**	e-M9:MPN/StHy (Sox14/Gpr101)
e-C3 (Penk/Hmcn1)	e-M10:MPN/StHy (Mc4r/Tac2)
e-C4 (Trh/Tac1)	**MPN posterior**
e-C5 (Npr3/Rgma)	e-N1:MPA/MPN (Pou3f2/Moxd1)
e-C6 (Npr3/Bnc2)	e-N2:MPA/MPN (Trh/Pamr1)
e-C7:BAC (Lhx9/Corin)	e-N3 (Crhr2/Neb)
e-C8 (Vglut2/Chst9)	e-N4 (Qrfprl/Sema3e)
e-H10 (Trhr/Egflam)	e-N5 (Tac1/Qrfpr)
e-N9 (Hmcn1/Crhr2)	e-N10:MPA/MPN (Pou3f2/Cbln1)
**PeFA**	e-N11 (Qrfpr/Gpr101)
e-F1:PeFA (Ucn3)	**PVN / SON**
**Thalamus**	e-P1:PVN/SON (Oxt)
e-T1:PVT (Shox2/Cck)	e-P2:PVN/SON (Avp)
e-T2:PVT (Shox2/Satb2)	e-P3:PVN (Crh/Trh)
e-H5 (Grik3/Itga8)	e-P4:PVN (Vglut2/Sst)

Inhibitory neuron types	
**BNST / MPN**	**AvPe / MnPO / LS**
i-B1:BNST (Aro/Cdh24)	i-X12 (Slc18a2/Prdm16)
i-B2:BNST (Moxd1/Nfix)	i-X13 (Grp/Prdm16)
i-B3:BNST (Tac2/Satb2)	i-X14:MPA/SHy (Bdnf/Nr4a2)
i-B4 (Satb2/Syt2)	**LPO**
i-B5:BNST (Satb2/Dlk1)	i-L1:PS/SHy/BNST (Cdh23/Adgrf5)
i-B6:BNST (Aro/Tac1)	i-L2:PS/SHy/BNST (Cartpt/Cdh23)
i-B10 (Satb2/Grp)	i-L7:LPO/PS/SHy (Foxp2/Lhx8)
i-B11 (Nr4a2)	**MPO**
i-B12 (Prdm16/Myo5b)	i-P1:MPA/SHy (Nts/Meis1)
i-B13:BNST (Igsf1/Dock5)	i-P2:MPA/SHy (Nmu)
**MPN / VMPO / VLPO**	**SCN / SBPV**
i-M1:MPN/StHy (Gal/Calcr)	i-S1:SCN (Avp/C1ql3)
i-M2:Pe/SHy (Gal/Th)	i-S2:SCN (Nms/Smo)
i-M3:Pe/VMPO (Kiss1/Th)	i-S3:SCN (Cck/C1ql3)
i-M4:Pe/VMPO (Nts/Sall1)	i-S4:SCN (Vip/C1ql3)
i-M5:MPN/StHy (Gal/Fbn2)	i-S5:SCN (Grp)
i-M6:VMPO (Calcr/Gpr101)	i-S6 (Calca/Onecut1)
i-M7:VLPO/MPN/VMPO (Gal/Gli3)	i-S7:SCN (Six3/Etl4)
i-M8:VLPO/MPN/VMPO (Cpne9/Cartpt)	**AHN / RCH / LHA / ZI**
i-M9:VLPO/MPN/VMPO (Rxfp1/Trpm8)	i-V9 (Th/Trh)
i-M10:LPO (Tac2/Cartpt)	i-X1 (Plch1/Cdh23)
i-M11:MPN/StHy (Pou6f2/Stxbp6)	i-X2 (Qrfpr/Col15a1)
i-M12:MPN/StHy (Pou6f2/Col9a1)	i-X3 (Onecut2/Gpr139)
i-X8 (Nr4a2/Gabrq)	i-X4 (Ttn/Onecut2)
i-X9 (Gpc4/Six3)	i-X5 (Prox1/Ebf1)
i-X10 (Onecut1/Ptger3)	i-X6 (Onecut2/Ddc)
**Periventricular**	**Thalamus**
i-S8:MPN/Pe(Lhx1/Satb2)	i-X15 (Pvalb/Glipr1)

bioRxiv preprint doi: https://doi.org/10.64898/2025.12.06.692770; this version posted December 8, 2025. The copyright holder for this preprint (which was not certified by peer review) is the author/funder, who has granted bioRxiv a license to display the preprint in perpetuity. It is made available under aCC-BY 4.0 International license.

Sullivan et al Figure S6

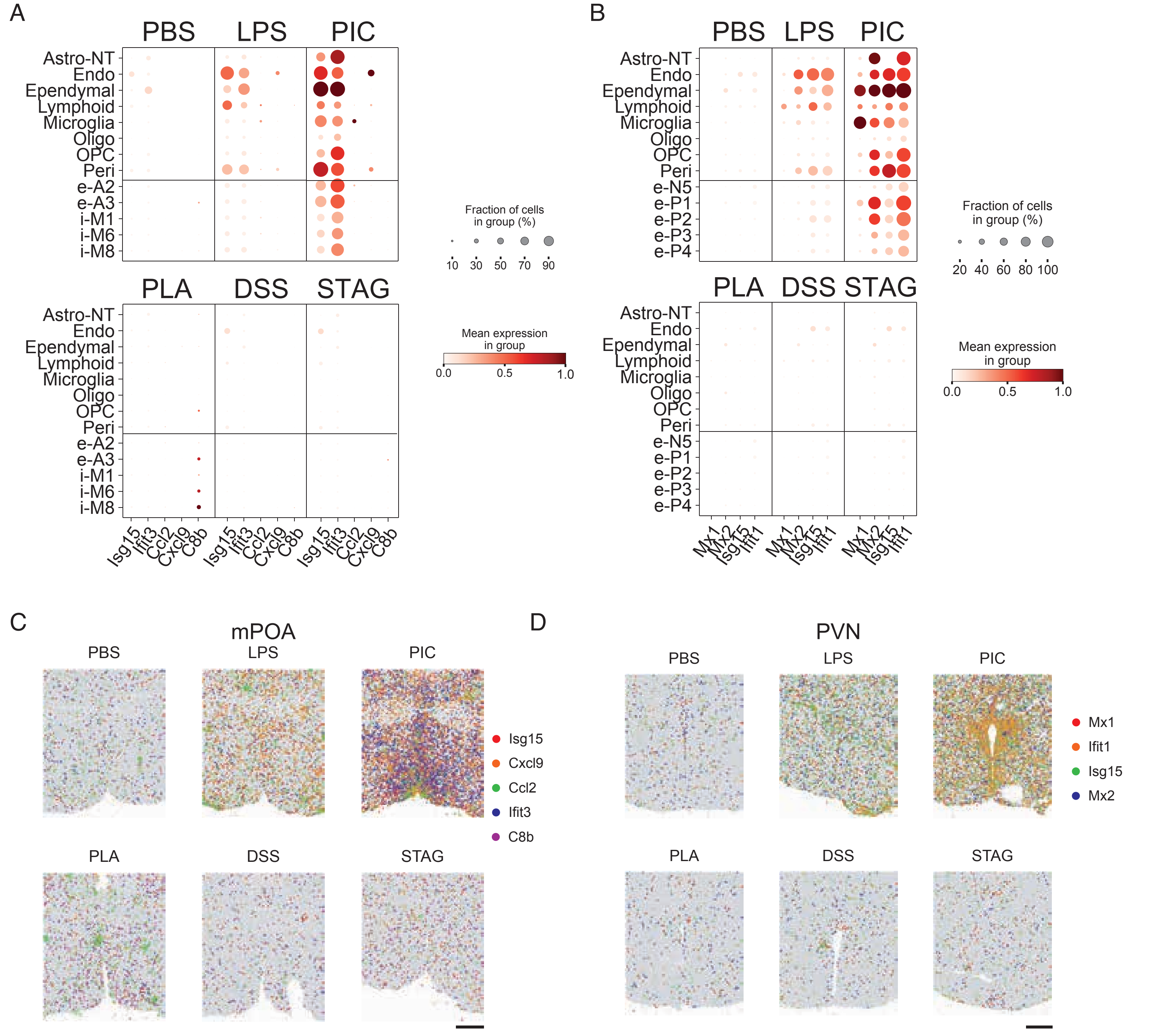

Supplement: Supplement 7 [file NIHPP2025.12.06.692770v1-supplement-7.pdf]
